# Montmorillonite–Rifampicin Nanohybrid for pH-Responsive Release of the Tuberculostatic

**DOI:** 10.3390/pharmaceutics15020512

**Published:** 2023-02-03

**Authors:** Elmar Damasceno Junior, Raquel de Melo Barbosa, Rita de Cássia Dantas da Silva, Felipe dos Santos Costa, Djalma Ribeiro da Silva, César Viseras, Luana Perioli, Nedja Suely Fernandes

**Affiliations:** 1 Institute of Chemistry, Federal University of Rio Grande do Norte, Natal 59078-970, RN, Brazil; 2 Pharmaceutical Technology Department, University of Granada, 18071 Granada, Spain; 3 Andalusian Institute of Earth Sciences, Spanish Research Council, University of Granada–CSIC-UGR, Avenida de las Palmeras 4, Armilla, 18100 Granada, Spain; 4 Department of Pharmaceutic Science, University of Perugia, 06123 Perugia, Italy

**Keywords:** tuberculosis, rifampicin, montmorillonite, nanohybrid, pH-responsive system

## Abstract

The present work describes the development of a hybrid and pH-responsive system for rifampicin using the clay mineral ‘montmorillonite’ as a nanocarrier. The influence of operational variables on the drug incorporation process was evaluated using 2^4^ factorial designs. Under optimized conditions, the experiment allowed an incorporated drug dose equivalent to 98.60 ± 1.21 mg/g. Hybrid systems were characterized by different characterization techniques (FTIR, XRD, TGA, DSC, and SEM) to elucidate the mechanism of interaction between the compounds used. Through in vitro release studies, it was possible to verify the efficacy of the pH-dependent system obtained, with approximately 70% of the drug released after sixteen hours in simulated intestinal fluid. The adjustment of the experimental release data to the theoretical model of Higuchi and Korsmeyer–Peppas indicated that the release of rifampicin occurs in a prolonged form from montmorillonite. Elucidation of the interactions between the drug and this raw clay reinforces its viability as a novel carrier to develop an anti-TB/clay hybrid system with good physical and chemical stability.

## 1. Introduction

Caused by the pathogenic agent *Mycobacterium tuberculosis*, tuberculosis (TB) is an infectious and highly transmissible disease that can affect not only the lungs (pulmonary TB) but also other organs of the body [1,2]. Despite being curable, until the arrival of the coronavirus (COVID-19) pandemic, TB was the leading cause of death from a single infectious agent worldwide. According to data from the World Health Organization (WHO), in 2021, about 10.6 million people were diagnosed with TB, an increase of 4.5% compared to 2020, and about 1.4 million people died as a result of the disease in the same year (WHO) [3].

The alarming data cited above is due to problems associated with the currently recommended therapeutic regimen, which consists of chemotherapy based on the administration of four antibiotics: isoniazid, rifampicin, pyrazinamide, and ethambutol [2,4,5]. These drugs are administered in high doses and for a prolonged period of time, which, depending on the patient’s response to treatment, can last up to 9–12 months, causing the appearance of serious side effects. Another worrying factor is the development of bacterial resistance to these antibiotics, which can lead to the emergence of multidrug-resistant TB. This problem has been declared a global health emergency by the WHO and may jeopardize disease control worldwide [1,2,5].

Rifampicin (RIF) is the main first-line drug used in the treatment of TB and is also used in multidrug therapy and the prophylaxis of leprosy. Despite having high bacteriostatic activity against *M. tuberculosis*, RIF has low bioavailability, mainly due to its low solubility and occurrence of polymorphism. Furthermore, RIF undergoes degradation in the acidic environment of the stomach, resulting in the formation of the degradation product 3-formyl rifamycin SV (3-FRSV). It can also undergo autoxidation, turning into rifampicin quinone. Therefore, it is necessary to administer high doses (approximately 10 mg/kg of the patient’s weight) for the drug to reach its desired therapeutic efficacy, causing the appearance of potentially toxic side effects, such as hepatitis, anorexia, skin eruptions, and renal failure [6,7,8,9].

Among the strategies reported in the literature to overcome these difficulties and improve the therapeutic efficacy of anti-TB drugs is to devise modified drug delivery systems (MDDS). Several works in the literature have reported obtaining MDDS for RIF, using the most diverse types of carriers, such as polymeric nanoparticles [10,11,12], microemulsions [13], dendrimers [14,15], hydrogels [6,16,17], liposomes [18], and lipid nanoparticles [19], among others. Despite being effective, such systems are based on expensive technologies that require the use of improved materials and/or devices. For TB, a disease that mainly affects the poorest of populations in underdeveloped countries, the search for MDDS that are effective, safe, and obtained from abundant and cheap materials is still necessary, since until recently, tuberculosis was considered a neglected disease [20].

Nanotechnology plays an important role in the development of new drug delivery systems. Systems based on nanoparticles allow delivery at lower doses, increase the solubility of the drug, improve its bioavailability, and thus minimize the side effects associated with its administration [21,22]. Inorganic nanoparticles (clay minerals, magnetic nanoparticles, silica, and others) have gained prominence in nanomedicine, mainly in disease diagnosis and drug delivery, due to their unique properties such as high stability, optical and magnetic properties, biocompatibility, biodegradability, and low cost [23,24].

Clay minerals are widely used in the pharmaceutical industry, as an active ingredient or excipient, in formulations for topical and oral use [25]. Recently, this type of material has attracted great interest from the scientific community for its potential application as a carrier of active substances. This interest is intrinsically linked to its physicochemical properties, such as adequate surface area, considerable adsorption capacity, and chemical inertness. In addition, clay minerals are biocompatible, abundant, and low-cost [26,27,28]. According to García-Villén et al. [29], clay minerals allow multiple pathways for interactions with microorganisms, decreasing water activity and cell adhesion, proliferation, and the formation of neotissues. All these characteristics make these nanoparticles viable to be explored in obtaining effective anti-TB drug delivery systems, making them promising candidates for improving therapeutic efficacy in disease treatment, including the treatment of multidrug-resistant infections [30].

Systems based on clay–drug nanohybrids have been widely reported in the literature, presenting interesting characteristics that have increased the bioavailability of several biomolecules, such as increased solubility of hydrophobic drugs, protection against degradation along the gastrointestinal tract, controlled release, and being pH responsive [20,31,32,33]. These systems are obtained through the establishment of interactions between the active sites of the nanocarrier and the functional groups present in the drug molecule. They can be prepared using several different methods, including: encapsulation, immobilization, ion exchange, and electrostatic interactions [33]. We have previously described how to obtain this type of system for the drugs isoniazid and rifampicin using the clay mineral palygorskite [4,20]. Other systems for anti-TB drugs, based on montmorillonite [34], halloysite [30,35,36], and palygorskite [1,37], have also been reported.

Montmorillonite (Mt) is a clay mineral of the 2:1 type and belongs to the smectite group. It has an organized structure in the form of layers, with a net negative charge between −0.2 and −0.6, resulting from random isomorphic substitution processes in the sheets of tetrahedra and octahedra. To balance this negative charge, Mt presents in its interlamellar region (between layers), exchangeable cations such as Na^+^, K^+^, Ca^2+^, and Mg^2+^, which are usually hydrated. This type of clay mineral swells easily, causing the formation of gels with well-defined rheological properties that exhibit a pseudo-plastic behavior. In addition, it has a cation exchange capacity (80–150 mEq/100 g) and a high specific area. All these characteristics make it possible for Mt to be applied in the development of new hybrid devices for modified drug delivery [38,39,40]. The ions present in the interlamellar region of montmorillonite are easily exchangeable for organic molecules, such as drugs, through cation exchange processes. Therefore, it is possible to obtain clay–drug hybrids through the intercalation of these bioactive molecules between the layers that make up the structure of the clay mineral [41,42]. In addition, Mt has surface silanol (Si-OH) groups that undergo different types of interactions with organic molecules, such as hydrogen bonds, ion–dipole interactions, coordination bonds, acid–base reactions, electrostatic attraction, and van der Waals interactions [43,44].

The main objective of this work is to obtain an Mt–RIF nanohybrid for the modified release of the anti-TB drug, as a way of overcoming the problems associated with its oral administration. As far as we know, this is the first work that reports the acquirement of this type of system for rifampicin that uses only montmorillonite as a carrier. The intercalation of the drug was evaluated through an experimental design, in order to investigate the influence of some variables in this process. The obtained hybrid was characterized by different techniques in order to further elucidate the clay–drug interaction. In vitro release studies were carried out to evaluate the performance of the obtained pH-responsive system.

## 2. Materials and Methods

### 2.1. Materials

Rifampicin was provided by NUPLAM-UFRN (Núcleo de Pesquisa em Alimentos e Medicamentos, Natal, Brazil) and supplied by the pharmaceutical company Sanofi, presenting a purity of 99.24%. The montmorillonite (Cloisite^®^ Na^+^, BYK Additives & Instruments, Wesel, Germany) used in the present work is of the sodium type—containing Na^+^ ions in the interlamellar region—and was provided by the company Colormix Especialidades (Barueri, Brazil). The other reagents used are analytical grade: ethyl alcohol (64-17-5, Dinâmica Química Contemporânea LTDA, Indaiatuba, Brazil), hydrochloric acid (37% p/p, 7647-01-0, Vetec, Duque de Caxias, Brazil), sodium hydroxide (1310-73-2, Vetec, Brazil), L-ascorbic acid (50-81-7, Labsynth, Diadema, Brazil), sodium chloride (7647-14-5, Vetec, Brazil), and monobasic sodium phosphate (10049-21-5, Dinâmica Química Contemporânea LTDA, Indaiatuba, Brazil).

### 2.2. Experimental Design

To evaluate the efficiency of drug incorporation into the clay mineral, a factorial design 2^4^ of the central composite design (CCD) type was used, consisting of sixteen experiments and three repetitions at the central point (mean level). The variables studied that could influence the RIF intercalation process in the Mt structure were as follows: Mt mass, RIF solution concentration, pH of RIF solution, and contact time. The volume of solution was fixed at 100 mL. The planning, estimation of the effects, and validation of the built model were obtained using Protimiza Experimental Design software (Campinas, Brazil). Table 1 presents the values used in the CCD for the four variables described above.

The incorporation occurred through an adsorption process and followed the methodology described by Oliveira, Alcântara, and Pergher [45], with some adaptations. Initially, the corresponding mass of Mt was weighed in a 250 mL Erlenmeyer flask. Then, 20 mL of deionized water was added. The mixture remained under constant agitation (180 rpm) at 20 ± 0.5 °C in an incubator for two hours to allow for the expansion of the clay mineral. 80 mL of a 40% (*v*/*v*) hydroethanolic solution of the drug was added, whose pH was adjusted by adding 1.0 mol/L HCl or 1.0 mol/L NaOH solutions. The solutions contained ascorbic acid at a concentration of 1 mg/mL to avoid RIF degradation during the experiments [4,46]. The flasks remained under agitation in an incubator, under the same conditions of rotation speed and temperature, for the pre-established period of time. At the end of each experiment, a 15 mL aliquot of the suspension was transferred to a Falcon tube and centrifuged for 15 min at 4000 rpm. The absorbance of each aliquot was determined using a UV/Vis spectrophotometer (UV-1800, Shimadzu, Kyoto, Japan) at 475 nm. Obtaining and validating the calibration curve followed the parameters in the ICH Harmonized Tripartite Guideline-Validation of Analytical Procedures [47]. The detection limit and quantification limit values obtained were 0.00023 and 0.00069 mg/mL respectively, indicating that the drug can accurately be detected and quantified by the technique, even at low concentrations [20]. The incorporated drug dose was determined by the equation below (Equation (1)) [48]:

(1) $$Incorporated\;dose\;drug\left(\frac{mg}{g}\right)=\frac{\left(C_{0}-C\right)\times V_{RIF}}{M_{Mt}}\times 1000$$

where *C*_0_ is the initial concentration of RIF (mg/mL), *C* is the concentration of RIF in solution (mg/mL) after the incorporation process, *V_RIF_* is the volume of RIF solution (mL), and *M_Mt_* is the mass of Mt (mg).

To validate the obtained model, the process was repeated under the optimized conditions in triplicate. The Mt–RIF nanohybrid was prepared under the same conditions. After adsorption, the powder was separated by vacuum filtration and dried in an oven at 60 °C for six hours. Then, the dried material was pulverized, sieved through a 100-mesh sieve, and stored in a desiccator for later use.

### 2.3. Characterization of Materials

The materials (RIF, Mt, and Mt–RIF) were characterized using X-ray diffraction (XRD), Fourier transform infrared spectroscopy (FTIR), thermogravimetry (TGA), and differential scanning calorimetry (DSC) techniques.

For structural characterization using the XRD technique, a Bruker D2 Phaser device (Karlsruhe, Germany) was used with the following specifications: CuKα radiation (λ = 1.54 Å), Ni filter, 0.02° step, 10 mA current, voltage 30 kV and Lynxeye detector.

FTIR spectra were obtained on a Shimadzu FTIR-8400S Iraffinity-1 spectrophotometer (Kyoto, Japan), with the following specifications: 32 scans, analysis range of 400–4000 cm^−1^, and resolution of 4 cm^−1^. Samples were prepared on KBr pellets.

TGA analyses were carried out on a Shimadzu TGA-50 thermobalance (Kyoto, Japan) using the following analysis conditions: platinum crucible, nitrogen as purge gas, gas flow rate of 50 mL/min, heating rate of 10 °C/min, and final analysis temperature of 900 °C.

DSC analyses for the samples were performed on a TA Instruments model Q20 instrument (New Castle, DE, USA). The analysis conditions were: gas flow rate of 50 mL/min, inert atmosphere (nitrogen gas), heating rate of 10 °C/min, and aluminum crucible. For all analyses, a mass of approximately 2 mg of sample was used.

Scanning electron microscopy (SEM) images were obtained using a TESCAN MIRA 4 scanning electron microscope (Brno–Kohoutovice, Czech Republic), using an in-beam SE secondary detector with 10 KeV energy. The samples were covered with gold film using a Denton Vacuum model Desk V vaporizer for 60 s and dispersed on carbon tape for high vacuum analysis.

### 2.4. In Vitro Release Studies and Release Kinetics

The in vitro release studies were carried out sequentially in four different dissolution media, in order to simulate the different pH values in the transit of a pharmaceutical form for oral use along the gastrointestinal tract. To simulate the conditions in the stomach region, the tests were performed for two hours in two different fluids: pH 1.2 (empty stomach) and pH 4.0 (‘fed’ stomach). Tests for simulated release in the small intestine were performed in a pH 6.8 fluid for four hours. Finally, to simulate the physiological conditions of the intestinal colon, a pH 7.4 fluid was used for ten hours [45,49,50].

All tests were carried out in a dissolving apparatus (model 800-2TS, Ethik Technology, São Paulo, Brazil), basket-type apparatus, at a constant speed of 100 rpm and temperature of 37 ± 0.5 °C. Five hundred micrograms of the Mt–RIF hybrid (approximately 50 mg of RIF) was encapsulated in size #00 gelatin capsules. Six hundred microliters of dissolution medium was used, maintaining the sink condition. After each time interval, 5 mL aliquots were removed, and subsequently, the withdrawn volume was replaced with fresh dissolution medium. The absorbance of each aliquot was determined using a UV/Vis spectrophotometer at 475 nm. Preparation of dissolution media followed the methodology described by Almeida et al. [50]. To prepare the dissolution medium at pH 1.2, 1 g of sodium chloride (NaCl) and 70 mL of concentrated hydrochloric acid (HCl) were dissolved in 1000 mL of deionized water. For the preparation of the pH 4 dissolution medium, 2.99 g of tribasic sodium acetate (NaC_2_H_3_O_2_•3H_2_O) and 1.66 mL of concentrated acetic acid (CH_3_COOH) were dissolved in 1000 mL of deionized water. The pH was adjusted using a 1.0 mol/L HCl solution. For the preparation of pH 6.8 dissolution medium, 0.3 g of sodium hydroxide (NaOH), 4 g of monobasic sodium phosphate (NaH_2_PO_4_•H_2_O), and 6.2 g of NaCl were dissolved in 1000 mL of deionized water. The pH 7.4 dissolution medium was prepared in the same way, with its pH adjusted by adding 1 mol/L NaOH. Ascorbic acid was added to each dissolution medium at a concentration of 1 mg/m to avoid oxidative degradation of RIF during release studies [46,51].

To evaluate the release mechanism, the experimental data were fitted to zero-order, first-order, Higuchi, and Korsmeyer–Peppas kinetic models. The general formulas of each model are shown in Table 2. 

## 3. Results

### 3.1. Experimental Design

The incorporation of the drug into the carrier structure is one of the great challenges in the design of delivery systems since several operational factors can influence this process [53]. According to Silva et al. [28], the intercalation process of organic molecules between Mt layers is governed by several variables, mainly concentration, temperature, contact time, and pH. Through the application of an experimental design, it was possible to evaluate the influence of some variables in the RIF incorporation process in Mt. Table 3 presents the planning matrix, with the experimental values of the incorporated drug dose (Y_1_) associated with each experiment. The experiments were performed at random, the last three corresponding to repetition at the central point.

According to Rodrigues and Iemma [54], the addition of central points enables the execution of statistical inference, without the obligation to perform repetitions of other experiments. Therefore, it is possible to calculate the residuals, the standard error and, consequently, carry out all the validation of the obtained model. Table 4 presents the results obtained with the application of the linear regression technique for the studied variables, considering a confidence level of 95% (significance level of 5%).

**Figure 1:**
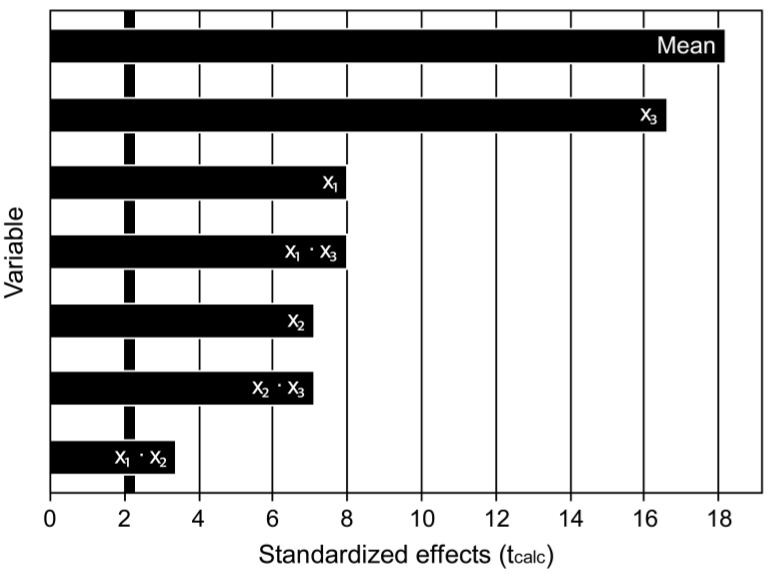
Pareto chart shows variables used in the process of the incorporation of RIF in Mt vs. the tabulated *t* value (standardized effects).

To be considered significant, the calculated effect (main or interaction) must have a *p*-value less than 0.05. Therefore, according to the results presented in Table 4, the main effects of the variables X_1_, X_2_, and X_3_, in addition to some interaction effects (X_1_•X_2_, X_1_•X_3_, X_2_•X_3_), are statistically significant and influence directly the incorporated drug dose values. This result can also be attested by the Pareto diagram (Figure 1), where the horizontal bars, arranged in descending order, represent the calculated absolute values of t, while the vertical line represents the tabulated t value (*p* = 0.05). In this case, the effect is considered significant when the calculated t value is greater than the tabulated t value, that is, when the horizontal bars exceed the vertical line highlighted in the graph [54,55].

**Figure 2:**
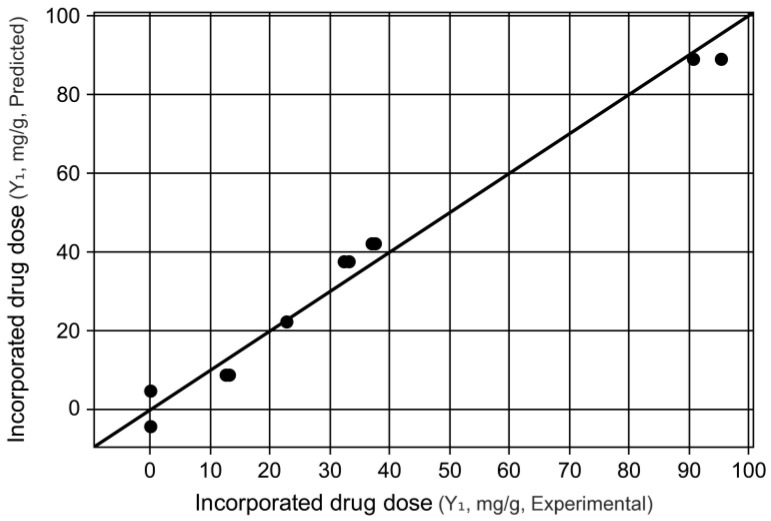
Graph of experimental values vs. predicted values of incorporated drug dose in mg/g.

After analyzing the effects, it was possible to obtain the empirical model that describes the relationship between the response (dependent variable) and the four operational variables studied. It should be noted that, in order to obtain this model, only the variables that had their effects considered significant in their coded values were considered. Equation (2) presents the first-order polynomial equation for the model. The adjustment of the theoretical model to the experimental data can be seen in the graph shown in Figure 2.

Y_1_ = 22.17 − 10.59 X_1_ + 9.45 X_2_ − 22.06 X_3_ − 4.51 X_1_ X_2_ + 10.59 X_1_ X_3_ − 9.45 X_2_ X_3_ (2)

In order to obtain the response surfaces and evaluate the influence of the variables studied in the process of drug incorporation into the clay mineral, it is necessary to validate the model obtained through analysis of variance (ANOVA). The results obtained are shown in Table 5.

From the results shown in Table 5, it appears that the regression was highly significant (*p* < 0.05). To statistically validate the model, it is necessary to analyze the calculated values for *F*, where the *F* associated with the regression must be greater than the tabulated *F* value, while the *F* associated with the lack of fit must be smaller than the tabulated *F* [54,55,56].

The tabulated *F* value for the regression, according to the degrees of freedom for the regression and for the residuals, considering a significance level of 5%, is 3.00. Therefore, we can attest that the model is statistically valid since the calculated value of *F* has a considerably higher value. Regarding the tabulated *F* for the lack of fit, considering the degrees of release associated with the lack of fit and pure error, its value, 6.01, is much lower than the calculated *F* value [55]. However, this result does not invalidate the model. According to Rodrigues and Iemma [54], cases like this occur when the lack of fit is low, but the standard error tends to zero. In addition, as seen in Figure 2, we can observe a certain normality in the adjustment of the model to the experimental data, in which there are no points that are very far from the line.

**Figure 3:**
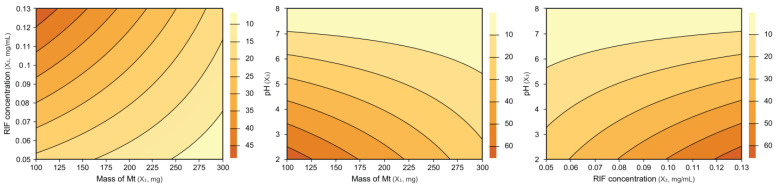
Response surfaces for comparison between the variables studied in the process of incorporating RIF in Mt.

Another factor to be analyzed is the value of the determination coefficient (R^2^). The regression model obtained in the present work presented a high value of R^2^, where 97.73% of the variations in the incorporated drug dose can be explained by the model, while only 2.27% of the variations cannot be explained. This result, considered quite satisfactory, also attests to the statistical validity of the obtained model [54,55,56]. Therefore, it is possible to obtain the response surfaces presented below (Figure 3). Such surfaces describe the influence of the studied variables on the response of interest which, in the present work, consists of the dose of the incorporated drug.

The darker the orange color, the higher the drug dose that is incorporated. Therefore, in general, based on the analysis of the response surfaces and the data presented in Table 4, the following observations can be reported:

- Variables X_1_ (mass of Mt) and X_3_ (pH) showed negative effects, indicating that higher incorporated drug dose values are obtained when the values of both variables were minimal.
- The variable X_2_ (RIF concentration) showed a positive effect, indicating that by increasing the RIF concentration in solution, the incorporated drug dose is maximized.
- The variable X_4_ (time) is not statistically significant and, therefore, does not have a considerable influence on the response of interest.
- To validate the previously presented results, the process was repeated in triplicate under the optimized conditions: Mt mass of 100 mg, RIF concentration of 0.125 and pH of 2. The incorporated dose was 98.60 ± 1.21 mg/g.

### 3.2. Characterization of Materials

#### 3.2.1. XRD

**Figure 4:**
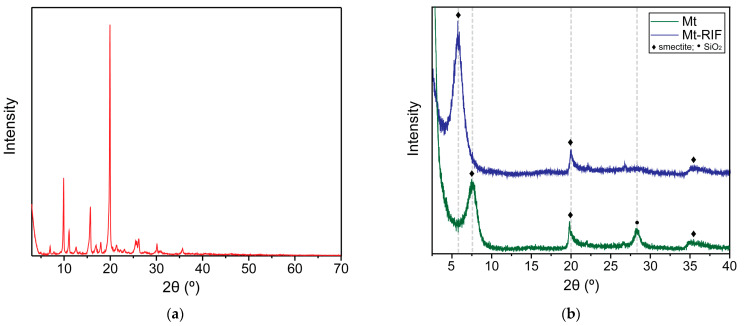
X-ray diffractogram of (**a**) RIF (in red), (**b**) Mt (in green) and Mt–RIF hybrid (in blue).

Figure 4 presents the X-ray diffractograms obtained for the materials. The RIF diffraction pattern (Figure 4a) is well discussed in work previously published by the group [4], showing reflections at 4.96, 7, 7.8, 10, 11.1, 12.7, 15.9, 16, 9, 18, 20, 21.4, 23.1, 26.2, 30.2, and 36° in 2θ. The diffractogram obtained for the pure Mt sample (Figure 4b) shows a peak at 7.54° in 2θ corresponding to the plane reflection (001), with a basal spacing value (d_001_) of 11.7 Å [45]. This spacing value is obtained by applying Bragg’s law [25]. When analyzing the diffraction pattern obtained for the Mt–RIF hybrid (Figure 4b), a displacement of the reflection from the (001) plane to lower angles by 2θ (5.65°) is observed, in comparison with the diffraction pattern of the pure mineral clay. Basal spacing d_001_ increased to 15.6 Å. Also observed is the disappearance of the reflection that occurs at approximately 28° in 2θ.

#### 3.2.2. FTIR

**Figure 5:**
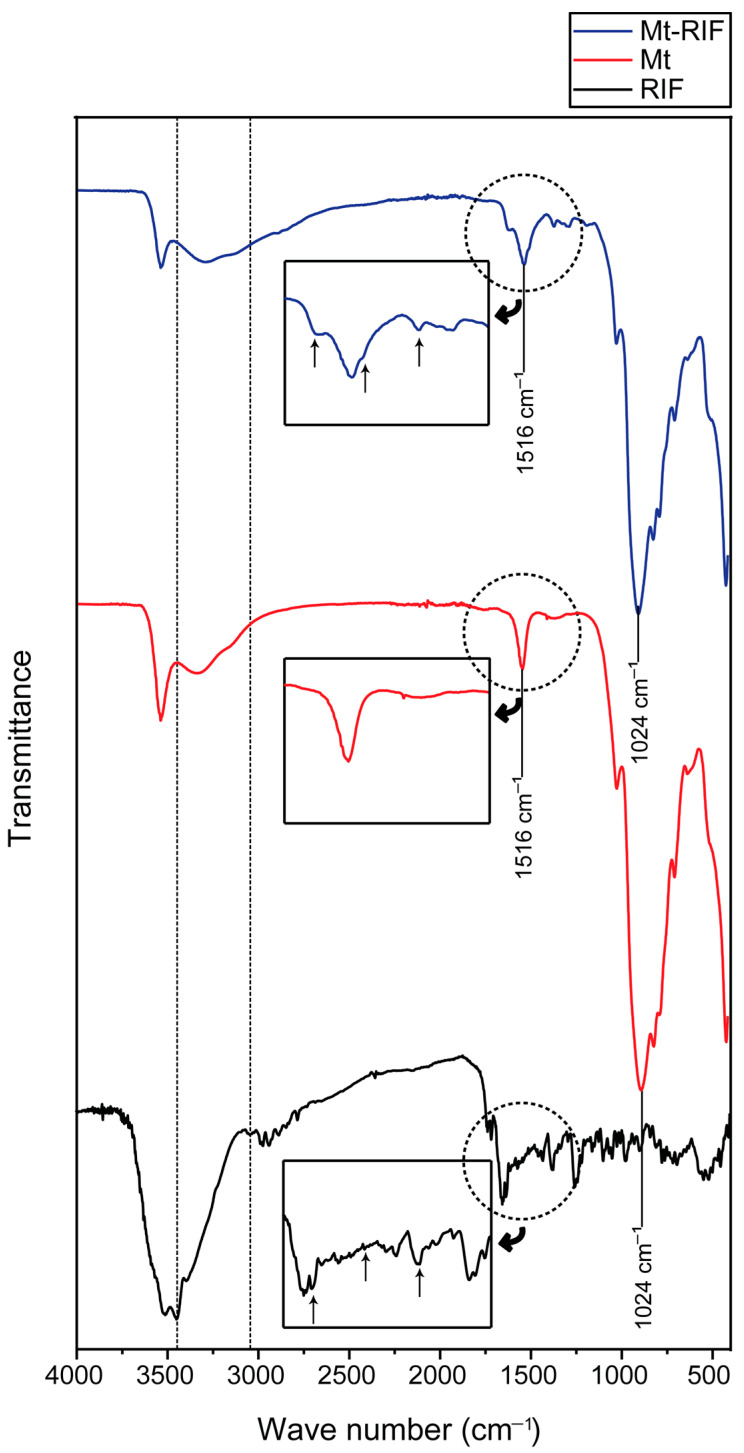
FTIR spectra for RIF (in black), Mt (in red) and Mt–RIF hybrid (in blue).

Figure 5 presents the FTIR spectra obtained for the materials. The RIF spectrum was well described in work previously published by the group [4]. From the infrared spectrum obtained for montmorillonite, it was possible to identify the main bands that characterize the material. The sharp band at 3540 cm^−1^ was attributed to OH stretching on Si–OH and Al–OH [48,57,58]. The broad absorption band between approximately 3450 cm^−1^ and 3210 cm^−1^ corresponds to the axial OH deformation of water molecules present in the interlamellar region and of water adsorbed on the surface [48,57,59,60,61]. The band at 1540 cm^−1^ corresponds to the OH vibrations of Si–OH [60,62]. The intense band at 1024 cm^−1^ is attributed to the Si–O bond [56,57,62]. The bands at 890 and 822 cm^−1^ correspond to the Si–O–Si, Al–O–Al, and Si–O–Al bonds. The band at 708 cm^−1^ is associated with bonds present in the octahedral layer of the clay mineral, such as Al–O–Al and Mg–O–Al [56].

Regarding the FTIR spectrum of the Mt–RIF hybrid (Figure 5), it was possible to identify all the bands present in the pure clay mineral spectrum, with small modifications. The band initially located between 3450 cm^−1^ and 3210 cm^−1^ becomes wider and less defined. The band at 3540 cm^−1^ became less intense, as did the band attributed to the Si–O bond at 1024 cm^−1^. It is also observed, from the highlight given in the figure, the appearance of small ‘shoulders’ at 1640 cm^−1^ and 1516 cm^−1^, close to the band corresponding to the OH vibrations of Si–OH at 1540 cm^−1^. Such bands, identified in the FTIR spectrum of the drug correspond, respectively, to the axial deformation of C=O of amide and to the angular deformation of N–H in secondary amines [4,63]. We also identify the appearance of subtle bands between 1380 and 1300 cm^−1^, attributed to C–O–C type bonds present in the RIF molecule (Figure 5) [4].

#### 3.2.3. Thermal Analysis

**Figure 6:**
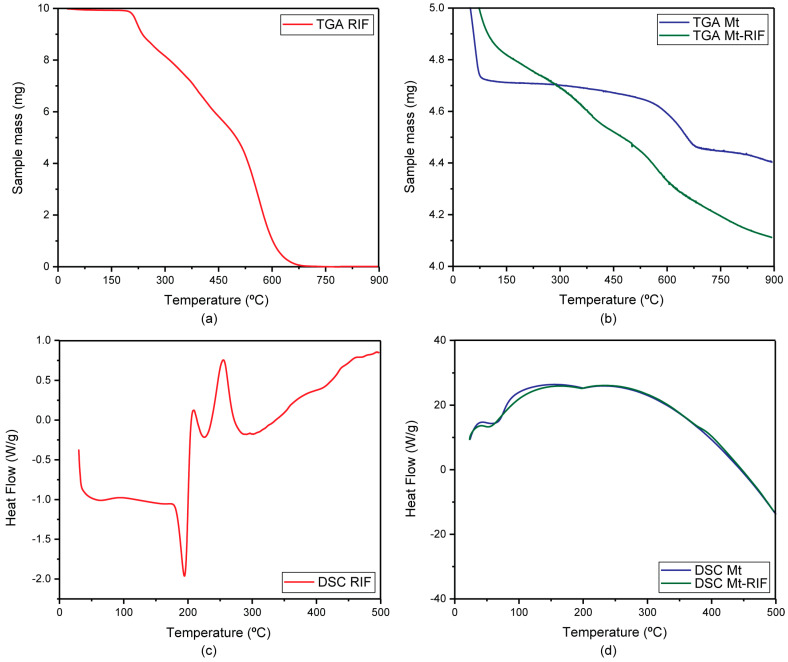
TGA curve for (**a**) RIF and (**b**) Mt (in blue) and Mt–RIF hybrid (in green). DSC curve for (**c**) RIF and (**d**) Mt (in blue) and Mt–RIF hybrid (in green).

Figure 6a shows the thermogravimetric curve obtained for the RIF. As already discussed in a previous work, three consecutive mass loss events are observed, referring to the thermal decomposition of the drug, between 190 °C and 300 °C, 300 °C and 450 °C, and 450 °C and 700 °C. Such events correspond to a mass loss of 18.0%, 24.6%, and 57.4%, respectively [4].

The DSC curve for rifampicin (Figure 6c) shows three thermal events. The first peak, which occurs in the range between 175 °C and 210 °C, is characterized as an endothermic event and corresponds to the drug melting process. The second event, evidenced by an exothermic peak that occurs shortly after the first, corresponds to the recrystallization process. According to the literature, during this process, RIF is converted from its polymorphic form II to its polymorphic form I. The heat released during recrystallization causes the thermal decomposition of the organic compound to commence. This decomposition proceeds with increasing temperature, as evidenced by the appearance of a new exothermic peak in the temperature range between, approximately, 225 °C and 290 °C [63,64].

From the analysis of the TGA curve obtained for montmorillonite (Figure 6b), it is possible to observe the occurrence of two stages of dehydration. The first, which occurs between 30 °C and 75 °C, corresponds to the loss of physically adsorbed water and water present in the interlamellar region. The second event, which occurs between approximately 300 °C and 750 °C, is associated with the dehydroxylation process (structural water loss) [39,61,65]. Both events showed a mass loss of 6%. The DSC curve (Figure 6d) shows a single endothermic peak, between 50 °C and 100 °C, corresponding to the first stage of dehydration [28].

#### 3.2.4. SEM

**Figure 7:**
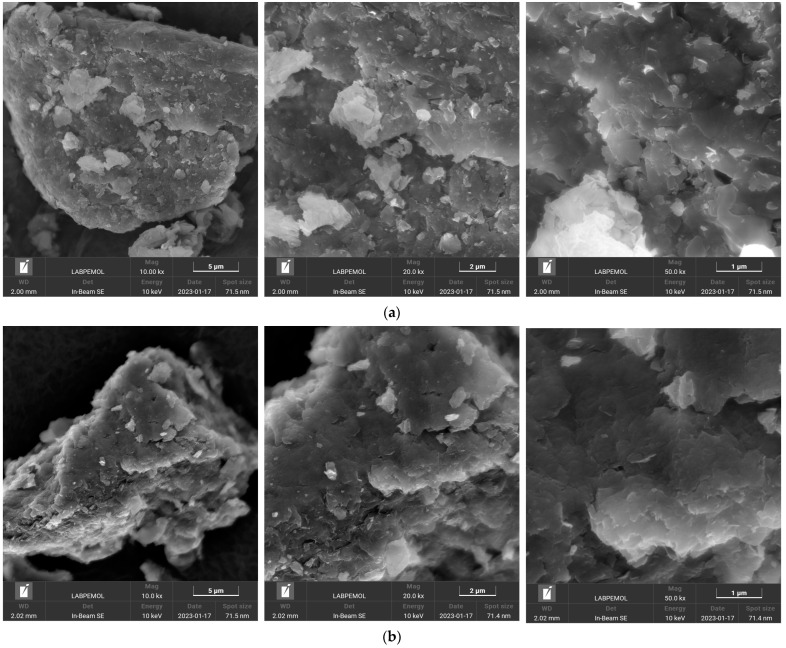
SEM images of (**a**) Mt and (**b**) Mt–RIF hybrid.

The SEM images obtained for the samples are presented in Figure 7. The Mt (Figure 7a) presented a morphology with aspects of leaves, typical of clay minerals of the smectite group. These leaves cluster together to form grains of larger dimensions [41,45,48]. The Mt–RIF hybrid (Figure 7b) presented similar morphology, where the appearance of leaves is also evident. There are no crystals related to the drug on the surface of the hybrid.

### 3.3. In Vitro Release Studies and Release Kinetics

**Figure 8:**
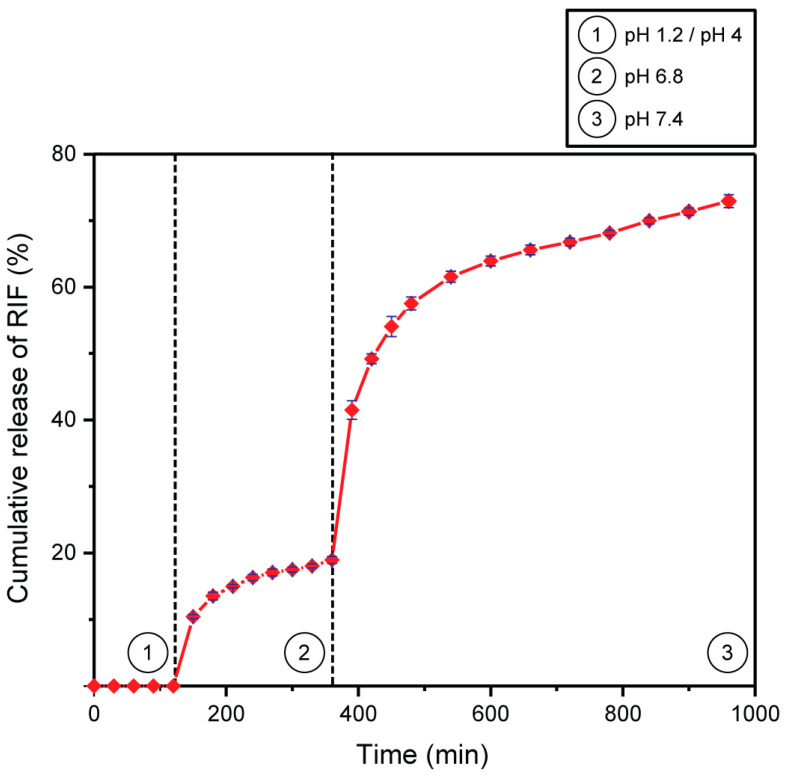
Cumulative release of RIF (mean ± standard deviation; *n* = 3).

Figure 8 shows the cumulative release profile of RIF from the Mt–RIF hybrid. As observed, there was no release in the simulated stomach fluid, both in the fasting condition (pH 1.2) and in the ‘full’ stomach condition (pH 4). It can be seen that the release process is extremely dependent on pH, occurring more efficiently as the pH of the medium increases (from pH 1.2/4 to pH 7.4). More than 70% of the dose was released in simulated intestinal fluid (pH 6.8 and 7.4). It is also observed that the system promoted a controlled release of RIF over sixteen hours. This time is, in real terms, even longer since, by the time limit of the tests carried out, 100% release had not yet been reached.

Table 6 presents the results of fitting kinetic models to experimental release data. As observed, it can be seen that at pH 6.8, the Higuchi and Korsmeyer–Peppas models were the ones that best fitted the data, with R^2^ values of 0.9840 and 0.9817, respectively. At pH 7.4, there was a better fit to the Korsmeyer–Peppas model (R^2^ = 0.9668).

## 4. Discussion

Through the experimental planning, it was possible to evaluate the influence of important parameters that affect the intercalation process of the drug in the structure of the clay mineral. In addition to minimizing the number of experiments, and consequently, reducing operating costs and time, this methodology allowed the construction of an empirical, statistically validated model that is capable of predicting the dose incorporated from the prior establishment of levels for the variables considered significant [54]. This model can also help in the design process of pediatric formulations, which generally require an adaptation of the incorporated drug dose.

As previously reported, isomorphic substitution processes occur in the tetrahedral and octahedral sheets of Mt, resulting in a net negative charge. To compensate for this charge, the structure of this clay mineral presents hydrated cations in the interlamellar region, which are easily exchangeable in a cation exchange process [27]. This is the physicochemical characteristic that supports the process of incorporation of drugs and other organic molecules into the Mt structure, which can be adsorbed in the interlamellar region or on the external surface. It can then be stated that the adsorption of RIF on Mt is a highly pH-dependent process, since the ionization state of the drug plays a significant role in the cation exchange process, directly impacting the performance of drug retention in the clay mineral [53,66]. Rifampin has two pK_a_ values. The first, 1.7, is related to the phenolic hydroxyl groups at C1, C4, and C8. The second value, 7.9, is associated with the nitrogen atoms of the piperazine ring [67]. According to the calculation presented in a previous work, RIF presents 100% of its molecules ionized at pH 2 due to the protonation of the nitrogen atoms in the piperazine ring. Thus, the drug exists predominantly in its cationic form (RIF+). At pH 6, there was no intercalation of the drug in the structure, since only 1.24% of the molecules were in the form of RIF+ [4]. This is why, as the pH of the drug solution decreases, the incorporated drug dose increases, as the cation exchange process actually takes place.

Similar results were presented by Afarani, Sarvi, and Alavijeh [68]. The authors studied the encapsulation process of vitamin B6 in montmorillonite. At pH 6.5, the vitamin was in its protonated form. Thus, its molecules began to occupy the interlamellar region and incorporation was governed by a cation exchange process. When the pH was increased to 9.5, the molecules were in their deprotonated form and began to interact with the edges of the clay mineral, which have a more negative charge. Bello et al. [69] studied the interaction of several drugs, all containing amine groups, with the sodium Mt structure. The authors demonstrated that the molecules in their protonated form replace sodium ions in the interlamellar region; therefore, the process is governed predominantly by cation exchange.

Regarding the mass, it was observed that the lower its value, the higher the drug dose that is incorporated. Mt has a high cation exchange capacity, being able to retain large quantities of organic molecules [45]. This result is interesting from an economic point of view, as it is possible to obtain higher incorporated drug dose values using a reduced amount of carrier. Similar results were found in works previously published by the group [4,20]. Belaroui et al. [70] also reported similar observations when evaluating the adsorption of an herbicide on palygorskite clay minerals. The authors showed that maximum adsorption capacity was reached with smaller masses of the adsorbent. Furthermore, as adsorbent mass increased, the amount of adsorbed herbicide tended to plateau.

Regarding the concentration, the response of interest is maximized when the concentration of RIF in solution is increased. This result is due to the fact that, when increasing the concentration, there is an increase in the driving force of mass transfer, facilitating the movement of molecules towards the active sites of the clay mineral [71,72]. Li et al. [48] studied the incorporation of dexibuprofen in pure montmorillonite and in acid-modified montmorillonite. In both cases, the drug load gradually increased with increasing concentration, until a saturation point was reached.

The solid-state characterization of the Mt–RIF hybrid is of paramount importance in proving the obtainment of the hybrid and in elucidating the interaction mechanism between the materials. As observed in the structural analysis by XRD, there is a displacement of the reflection (001) from Mt to smaller angles in 2θ, with an increase in the basal spacing d_001_, which went from 11.7 Å to 15.6 Å. This result clearly indicates the intercalation of RIF molecules in the interlamellar region of Mt. Similar results were reported in works in the literature that studied the intercalation of other molecules in the montmorillonite structure [28,29,48,66,69]. Also, the diffraction peak becomes more intense and defined. According to García-Villén et al. [29], this result is an indication that the structure of the nanohybrid is highly ordered. The disappearance of the reflection at 28° in 2θ may be associated with the process of Mt dealumination, caused by the addition of acid used in the pH regulation of the RIF solution. This process is characterized by the leaching of octahedral cations and the formation of amorphous silica, leading to partial damage to the clay mineral structure. This favors the formation of pores, and consequently increases the drug loading capacity [48,73]. The absence of peaks referring to the crystalline structure of RIF in the Mt-RIF diffraction pattern (Figure 4b) indicates amorphization of the drug during the cation exchange process, in addition to confirming the absence of RIF crystals on the surface of the nanohybrid [29,48]. Such observations are also confirmed by DSC and SEM analysis.

The FTIR analyses indicated the functional groups involved in the interaction process between the materials. By analyzing the spectrum obtained for the Mt–RIF hybrid, it is observed that there was no change in the positions of the bands, suggesting that the adsorption process occurs preferentially in the interlamellar region, with weak interaction on the outer surface [74]. According to Silverstein et al. [57], the increase/decrease in band intensity or splitting are indications of interaction between materials. Therefore, the changes in certain bands observed in the spectrum obtained for the nanohybrid suggest the establishment of hydrogen bond-type interactions between the nitrogen atoms of the RIF molecule and the OH of the water present in the interlamellar region [73]. Such results indicate the coordination of drug molecules to silanol (Si-OH) groups on the surface, confirming the effective inclusion of RIF molecules in the interlamellar region of Mt, with drug solubilization in the carrier structure [35,75].

From the TGA curve obtained for the Mt–RIF hybrid (Figure 6b), it was possible to confirm the presence of RIF in the structure. It is observed that the first mass loss, referring to the first stage of Mt dehydration, was smaller, corresponding to approximately 3%. Soon after, a mass loss corresponding to 15% of the sample begins in the temperature range between 15 °C and 750 °C. This event corresponds to the thermal degradation of the incorporated RIF, followed by the Mt dehydroxylation process. In the DSC curve (Figure 6d), only the endothermic peak referring to the first dehydration of Mt is observed, without the appearance of the peak referring to the melting of RIF. The absence of this peak indicates a change in the crystalline form of RIF after interaction with the clay mineral, as confirmed by XRD analysis [28].

From the SEM images obtained for the Mt–RIF hybrid (Figure 7b), we can see that the drug incorporation process does not change the morphology of the nanocarrier. The absence of drug crystals on the surface corroborates the results obtained by the other characterization techniques, indicating an amorphization of RIF and good homogenization of the drug in the nanocarrier structure [48].

The in vitro release studies demonstrated the ability of montmorillonite to protect the drug molecules from degradation in the stomach environment since there was no release in conditions that simulated this environment. In this case, the drug molecules remain in the form of RIF+, occupying the interlamellar region of Mt. According to Damasceno-Junior et al. [4], at acidic pH values, the oxygen atoms of the silanol groups and water molecules act as bond acceptors, while the protonated nitrogens of the piperazine ring act as donor atoms. Therefore, as oxygen is more electronegative, the hydrogen bond established between materials is stronger [50]. In the intestinal environment, RIF molecules are deprotonated, being gradually replaced by cations present in the dissolution fluid through a process governed by the cation exchange mechanism [45]. The hydrogen bond becomes weaker since the nitrogen atoms, which are less negative, start to act as acceptors [4,20].

The results demonstrate that montmorillonite can, in this specific case, be used alone as a carrier, without the need for modification or combination with other materials (obtaining a composite). The system was able to protect the drug from the degradation suffered by RIF in the stomach, demonstrating that the hybrid increases drug stability. In addition, RIF was released in a controlled manner in the simulated intestinal fluid, the preferential environment for the absorption of orally administered drugs [75]. Such results promote a significant improvement in the bioavailability of RIF, meaning that the patient would need a lower dose for therapeutic efficacy to be achieved. The prolonged-release also makes it possible to reduce the frequency of dosing, thereby minimizing or eliminating any associated side effects [76,77,78]. The *n* exponent values obtained by adjusting the Korsmeyer–Peppas model were 0.275 and 0.268 for pH 6.8 and pH 7.4, respectively, characterizing a release that can be described as a diffusion process based on Fick’s law (*n* < 0.5) [39,52,79]. These results indicate that the RIF release process is diffusion-controlled, whereas Higuchi’s model also describes the release as a diffusion process, without matrix erosion or swelling [79]. The cations present in the release medium diffuse, promoting cation exchange with the drug molecules present in the interlamellar region [48].

## 5. Conclusions

The main objective of this work was to obtain a nanohybrid for the tuberculostatic rifampicin using montmorillonite as a nanocarrier. Initially, the incorporation process was evaluated through an experimental design, in order to investigate the influence of some variables on the intercalation of the drug in the structure of Mt. From the results obtained, it can be concluded that the process is extremely pH dependent, with higher incorporated drug dose values obtained at pH 2. The interaction process occurs by cation exchange, with the protonated drug molecules occupying the interlamellar region.

The obtained nanohybrid was characterized by different techniques, and the interaction mechanism between the materials was elucidated. Through XRD analysis, effective intercalation of the drug in the clay mineral structure was confirmed. The FTIR spectra indicated that the formation of hydrogen bonds occurs between the nitrogen atoms of the piperazine ring of RIF and the OH of water molecules and surface silanol (Si–OH) groups. Thermal analysis confirmed the RIF amorphization process. This process favored the increase in drug solubility observed in the results obtained through in vitro release tests.

Finally, it can be concluded that the Mt–RIF nanohybrid was able to increase the stability of RIF along the gastrointestinal tract, releasing the drug in a controlled and pH-responsive manner in simulated intestinal fluid (pH 6.8 and 7.4). The system provides an increase in the bioavailability of RIF, minimizing the problems associated with its oral administration. The experimental release data fit better with the Higuchi and Korsmeyer–Peppas models, indicating that the release is mainly controlled by diffusion and that this diffusion follows Fick’s law.

## Figures and Tables

**Table 1:** Values used in the CCD to evaluate four operational variables for the evaluation of the RIF incorporated process in Mt.

Variable Name	−1	0	+1
Mass of Mt (mg, X_1_)	100	200	300
RIF concentration (mg/mL, X_2_)	0.05	0.09	0.13
pH (X_3_)	2	5	8
Time (X_4_)	6	15	24

where ‘−1’ and ‘+1’ correspond to the lower and upper levels, respectively, and ‘0’ to the mean level (central point), associated with each variable. The response (dependent variable) evaluated was the ‘Incorporated Drug Dose (mg/g)—Y_1_’.

**Table 2:** Kinetic models used in the evaluation of the RIF release mechanism from the Mt–RIF nanohybrid [52].

Kinetic Model	Equation ^1^	Plotted Graph
Zero order	*Q_t_* = *Q*_0_ + *K*_0_*t*	*Q_t_* versus *t*
First order	log *Q_t_* = log *Q*_0_ + *K*_1_*t*	log *Q_t_* versus *t*
Higuchi	*Q_t_* = *K_H_t*^1/2^	*Q_t_* versus *t*^1/2^
Korsmeyer–Peppas	*M_t_*/*M*_∞_ = *Kt^n^*	log (*M_t_*/*M*_∞_) versus log *t^n^*

^1^ *Q_t_*—amount of drug released at time *t*; *Q*_0_—initial amount of drug in solution; *M_t_*/*M*_∞_—cumulative drug release; *K*_0_, *K*_1_, *K_H_*, *K*—characteristic constants of each model; *t*—time, *n* is an exponent that characterizes the mechanism of release of the RIF.

**Table 3:** Response values obtained for evaluating the process of incorporation of RIF in Mt using experimental design 2^4^ of the CCD type.

Experiment	Mass of Mt (mg, X_1_)	RIF Concentration (mg/mL, X_2_)	pH (X_3_)	Time (h, X_4_)	Incorporated Drug Dose (mg/g, Y_1_)
1	100	0.05	2	6	37.65
2	300	0.05	2	6	12.8
3	100	0.125	2	6	95.5
4	300	0.125	2	6	33.3
5	100	0.05	8	6	0
6	300	0.05	8	6	0
7	100	0.125	8	6	0
8	300	0.125	8	6	0
9	100	0.05	2	24	37.1
10	300	0.05	2	24	13.33
11	100	0.125	2	24	90.9
12	300	0.125	2	24	32.33
13	100	0.05	8	24	0
14	300	0.05	8	24	0
15	100	0.125	8	24	0
16	300	0.125	8	24	0
17	200	0.0875	5	15	22.75
18	200	0.0875	5	15	22.75
19	200	0.0875	5	15	22.75

**Table 4:** Estimated regression coefficients and corresponding *t* and *p* values obtained during the central composite design for the RIF incorporation process in Mt.

Name	Coefficient	Standard Error	Calculated *t*	*p*-Value
Mean	22.17	1.22	15.03	0.0000
X_1_	−10.59	1.33	−6.59	0.0002
X_2_	9.45	1.33	5.88	0.0004
X_3_	−22.06	1.33	−13.72	0.0000
X_4_	−0.35	1.61	−0.22	0.8334
X_1_•X_2_	−4.51	1.61	−2.81	0.0230
X_1_•X_3_	10.59	1.61	6.59	0.0002
X_1_•X_4_	0.29	1.61	0.18	0.8592
X_2_•X_3_	−9.45	1.61	−5.88	0.0004
X_2_•X_4_	−0.35	1.61	−0.22	0.8345
X_3_•X_4_	0.35	1.61	0.22	0.8334

**Table 5:** ANOVA for the answer (Y_1_).

Variation Source	Sum of Squares	Degrees of Freedom	Mean Square	F_calc_	*p*-Value
Regression	14,551.9	6	2425.3	86.1	0.0000
Residuals	337.9	12	28.2	-	-
Lack of fit	337.9	10	33.8	Infinity	NaN
Pure error	0.0	2	0.0	-	-
Total	14,889.8	18	-	-	-
R^2^ = 97.73%					

**Table 6:** Kinetic models used in the evaluation of the RIF release mechanism from the Mt–RIF nanohybrid.

Kinetic Model	R^2^	
	pH 6.8	pH 7.4
Zero order	0.9356	0.8443
First order	0.8976	0.7486
Higuchi	0.9840	0.9384
Korsmeyer–Peppas	0.9817	0.9668
